# Anti-Saccades in Cerebellar Ataxias Reveal a Contribution of the Cerebellum in Executive Functions

**DOI:** 10.3389/fneur.2018.00274

**Published:** 2018-04-23

**Authors:** Elena Pretegiani, Pietro Piu, Francesca Rosini, Pamela Federighi, Valeria Serchi, Gemma Tumminelli, Maria Teresa Dotti, Antonio Federico, Alessandra Rufa

**Affiliations:** ^1^Eye-Tracking and Visual Application Laboratory (EVALab), Department of Medicine, Surgery and Neurosciences, University of Siena, Siena, Italy; ^2^Laboratory of Sensorimotor Research, National Eye Institute, National Institutes of Health, Department of Health and Human Services, Bethesda, MD, United States; ^3^Neurological and Neurometabolic Unit, Department of Medicine, Surgery and Neurosciences, University of Siena, Siena, Italy; ^4^Department of Business and Law, University of Siena, Siena, Italy

**Keywords:** frontocerebellar network, goal-directed actions, cognitive functions, latency, error

## Abstract

**Objective:**

Increasing evidence suggests a cerebellar contribution to modulate cognitive aspects of motor behavior and executive functions. Supporting findings come from studies on patients with neurodegenerative diseases, in which however, given the extent of the disease, the specific role of the cerebellum, could not be clearly isolated. Anti-saccades are considered a sensitive tool to test executive functions. The anti-saccade underlying neural network, consisting of different cortical areas and their downstream connections including the lateral cerebellum, has been largely clarified. To separate the role of the cerebellum with respect to other cortical structures in executive control, we compared the anti-saccade performances in two distinct cohorts of patients with cerebellar disorders (with and without cerebral cortical involvement).

**Methods:**

Eye movements during the execution of anti-saccades were recorded in 12 patients with spinocerebellar ataxia type 2 (a cortical-subcortical neurodegenerative disease), 10 patients with late onset cerebellar ataxia (an isolated cerebellar atrophy), and 34 matched controls.

**Results:**

In the anti-saccade task, besides dynamic changes already demonstrated in the pro-saccades of these patients, we found in both groups of cerebellar patients prolonged latency with larger variability than normal and increased directional error rate. Errors, however, were corrected by cerebellar patients as frequently as normal. No significant differences were found in patients with and without cortical involvement.

**Conclusion:**

Our results indicate, in a large cohort of cerebellar patients, that the cerebellum plays a critical role in the regulation of executive motor control not only, as well known, by controlling the end of a movement, but also modulating its initiation and reducing reflexive responses that would perturb voluntary actions.

## Introduction

The cerebellum plays a well-known role in adapting motor responses by controlling the gain of a movement, its accuracy preservation, and by learning from endpoint errors (1–4). In the motor system, adaptive behavior is considered the result of cerebellar internal models. These models predict the sensory consequences of an action by integrating an efference copy of its motor command (forward model). A growing number of studies, however, have suggested that cerebellar internal models might act not only in the motor but also in the sensory, behavioral, and cognitive domains (5–11). These observations support the theory that the cerebellum plays a central role in monitoring executive brain performances (12, 13). Nevertheless, to which extent and for which functions this theory is valid, and which cerebellar areas are involved, remains largely unclear.

The anti-saccade task (14) is considered a sensitive tool to test executive functions and provides an effective way of studying higher-level motor control. It requires the cancelation of a reflexive saccade toward a suddenly appearing target (pro-saccade) and the execution of a self-placed movement toward a mirrored non-marked position (anti-saccade). Moreover, if a direction error is made (unsuppressed pro-saccade toward the target), a corrective anti-saccade is demanded. The neural network underlying anti-saccades, also important in the generation of pro-saccades, has been partially clarified and consists of different areas in the medial and lateral frontal cortex, the parietal cortex, the basal ganglia, and the cerebellum (15–23). Frontal lobe dysfunctions have been associated with a global disruption of the anti-saccade planning, resulting in increased latency and error rate and inability to correct errors (15).

Similar abnormalities, however, have recently been reported also in cerebellar patients. A few studies, indeed, have found increased directional error rate and prolonged latencies of anti-saccades in patients with hereditary cerebellar ataxia (24–27), focal cerebellar lesions (28), and acquired degenerative cerebellar diseases (29–31). Therefore, while the medial cerebellum, dorsal vermis, and fastigial nucleus [oculomotor vermis (OMV)], is traditionally considered the structure where saccade dynamic is regulated online (32), the cerebellar hemispheres and dentate nuclei (lateral cerebellum), conventionally not considered part of the oculomotor system, have been proposed to play a role in monitoring high-level saccadic behaviors, including the anti-saccades (24, 32–37).

The control of executive function, however, remains largely considered a frontal cortex activity, with the cerebellum having only a possible ancillary contribution. Unfortunately, most studies on anti-saccades in neurodegenerative cerebellar ataxias enrolled patients with involvement of other cerebral structures beyond the cerebellum. These studies, thus, did not help to disambiguate the cortical vs cerebellar source of impaired anti-saccade performance, leaving unresolved the question of the exact role of the cerebellum in the regulation of voluntary movements. Patients with cerebellar stroke, on the other side, have often confined lesion, but the acute dynamic of the damage might lead to different dysfunction and compensatory mechanism than those resulting from slowly degenerative diseases.

Moreover, most anti-saccadic studies on cerebellar patients have reported just the directional error frequency and the anti-saccadic latency, but not the frequency and latency of corrective saccades. Reduced frequency of correction has been recently related with cortical involvement (38) and could be used to better distinguish the cerebral vs cerebellar contribution.

Therefore, we aimed to segregate the cerebellar vs cerebral contribution to voluntary movement by comparing the anti-saccade performances of two homogeneous cohorts of patients with different cerebellar diseases: late onset cerebellar ataxia (LOCA) and spinocerebellar ataxia type 2 (SCA2). LOCA is a “pure cerebellar syndrome,” usually sporadic (39), in which the neurodegenerative process is confined to the cerebellum, while autosomal dominant SCA2 (MIM 183090) is caused by the expansion of CAG repeats within the *ATXN2* gene (MIM 601517) and it is clinically characterized by a “cerebellar-plus syndrome” (40, 41), where cerebellar atrophy is associated with cerebral cortical atrophy and brainstem involvement (42).

To better clarify the cerebellar influence to executive functions, we considered not only the anti-saccadic directional error rate and latency, but also the frequency of correction of the directional errors and the intersaccadic interval between erroneous pro-saccades and corrective anti-saccades. The anti-saccadic parameters were compared with those of 34 controls and correlated with disability scores.

We hypothesized that if the cerebellum plays just an ancillary role in executive control, with respect to other cortical areas (i.e., the frontal lobes), performances should be worse in the patients with both cerebellar and cerebral involvement than just isolated cerebellar atrophy. Conversely, if the cerebellum plays a limiting step in executive function, performances should be similarly affected in cerebellar patients with and without cortical involvement.

We found impaired increased directional error rate (with normal corrective frequency) in all cerebellar patients without significant differences between those with and without cortical involvement. Our study, thus, demonstrates in a large cohort of cerebellar patients, a crucial contribution of the cerebellum in managing executive functions by suppression of reflexive movements. Moreover, since the latency of anti-saccades in SCA2 and LOCA patients was significantly longer and variable than in controls, but not significantly different between the two groups, we suggest that the cerebellum is also involved in monitoring voluntary motor performance by controlling the onset of saccades until the ambiguity on its planning is resolved.

## Materials and Methods

### Patients and Controls

Clinical and magnetic resonance imaging (MRI) data of patients are resumed in Table 1. Twelve patients with genetically confirmed SCA2 (9 males, 3 females), mean age 38.7 years (range 25–53 years) and mean age at onset 30.5 years (range 17–44 years); and 10 patients with LOCA (5 males, 5 females), mean age 42.4 years (range 27–53 years), and mean age at onset 36.9 years (range 20–52 years) were enrolled in the study. All patients underwent a complete neurological, neuro-ophthalmological, and International Cooperative Ataxia Rating Scale (ICARS) (43) examination. All patients also underwent a structural brain MRI, evaluated separately by two experienced neuroradiologists, and the recommended clinical protocol for ataxias, including pertinent genetic and laboratory testing. In patients with LOCA, known causes of acquired and inherited ataxias were ruled out (Friedreich ataxia, fragile X-premutation, ataxia-telangiectasia, ataxias associated with mutation of aprataxin, senataxin or sacsin, metabolic causes of ataxia such as abetalipoproteinemia, vitamin E deficiency, late-onset Tay-Sachs disease, cerebrotendinous xanthomatosis, autosomal dominant spinocerebellar ataxias due to *de novo* mutations). Only two SCA2 patients (old relatives of two patients included in the study) with qualitatively severe cortical atrophy and cognitively compromised (MMSE <23) were excluded from the study. The control group consisted of 34 healthy age-matched subjects (13 males, 21 females) of mean age 36 years (range 19–65 years). All subjects gave their informed consent. The study respected the Declaration of Helsinki and was approved by the local Ethics Committee.

**Table 1:** Clinical, genetic, and magnetic resonance imaging (MRI) findings of spinocerebellar ataxia type 2 (SCA2) and late onset cerebellar ataxia (LOCA) patients.

Subject	CAG repeats	ICARS	MRI
SCA2-1	43	26/100	Severe brainstem and cerebellar atrophy
SCA2-2	38	36/100	Mild cortical moderate to severe brainstem and cerebellar atrophy
SCA2-3	36	59/100	Severe brainstem and cerebellar atrophy
SCA2-4	39	42/100	Severe brainstem and cerebellar atrophy
SCA2-5	37	15/100	Moderate brainstem and cerebellar atrophy
SCA2-6	40	30/100	Moderate to severe brainstem and cerebellar atrophy
SCA2-7	42	56/100	Mild cortical, moderate to severe brainstem, and cerebellar atrophy
SCA2-8	35	36/100	Moderate brainstem and cerebellar atrophy
SCA2-9	36	35/100	Severe brainstem and cerebellar atrophy
SCA2-10	38	37/100	Pons and cerebellar atrophy
SCA2-11	40	23/100	Pons and cerebellar atrophy
SCA2-12	41	48/100	Severe brainstem and cerebellar atrophy. Mild cortical atrophy
LOCA-1		28/100	Global cerebellar atrophy
LOCA-2		40/100	Global cerebellar atrophy
LOCA-3		35/100	Global cerebellar atrophy
LOCA-4		25/100	Global cerebellar atrophy
LOCA-5		43/100	Global cerebellar atrophy
LOCA-6		27/100	Global cerebellar atrophy
LOCA-7		27/100	Global cerebellar atrophy
LOCA-8		34/100	Global cerebellar atrophy
LOCA-9		41/100	Global cerebellar atrophy
LOCA-10		38/100	Global cerebellar atrophy

*The table shows the scores of the International Cooperative Ataxia Rating Scale (ICARS), and the MRI outcomes for the LOCA and SCA2 patients. ICARS is a specifically validated scale, in the interval from 0 to 100, to evaluate cerebellar ataxia, with higher values denoting major clinical anomalies. For the SCA2 patients, the number of trinucleotide repeats (CAG) is also reported*.

### Eye Movement Recording

Eye movements were recorded with an ASL 504 eye-tracker device (Applied Science Laboratories, Bedford, MA, USA). Data acquisition and visual stimulation were controlled by a PC (3 GHz Pentium) running a custom software dedicated to real-time data acquisition. Eye position was sampled at 240 Hz, digitized with a resolution of 16 bits, corresponding to a sensitivity of recorded eye position of 0.16 deg, and stored for off-line analysis. The visual stimulus was a red dot (luminance 63 cd/m^2^) with a diameter subtending a visual angle of 0.4 deg, presented on a black background (luminance 2.5 cd/m^2^). We used a 310 × 510 mm LCD screen, with a resolution of 1,024 × 768 pixels, positioned 720 mm from the subject’s eyes.

The subjects were seated in a darkened room while their head movements were minimized by a chinrest with a bite bar. Each recording session was preceded by an interactive calibration procedure based on nine static points and three static points of validation.

### Anti-Saccade Task

Each anti-saccade task consisted in a block of 40 trials per session, with a total of 80 trials. During each trial, a central fixation point was presented for 500 ms. After the disappearance of the central fixation point, a peripheral target was presented randomly with respect to direction (left, right) and position (10, 18 degrees) for 2,500 ms. We chose two different target eccentricities (10 and 18 degrees) since possible different neural networks might control smaller and larger saccades (44).

Subjects were instructed to make a mirror saccade to the opposite direction of the target. To reduce memory engagement during the task, the correct anti-saccade position was unmarked after the anti-saccade landing, and it was followed by the appearance of the central fixation point. At this moment, the subject was instructed to return to the center of the screen. The trials were obtained in two different sessions (same day or the day after) depending on the patient’s compliance. All trials were analyzed by two expert neurologists (Francesca Rosini and Gemma Tumminelli).

### Signal Processing

Signal processing was conducted off-line using a semi-automatic detection algorithm. Data were filtered using a third-order Butterworth low-pass digital filter with −3 dB attenuation at 25 Hz cut-off frequency. A velocity threshold of 10 deg/s was used to determine the starting and ending times of saccades. Eye velocity was obtained with an eight-point central difference derivative algorithm having a bandwidth larger than 70 Hz at a digitization frequency of 240 Hz (45). Saccades with latency less than 100 ms, were considered anticipatory and excluded. We excluded all oblique saccades whose direction had an angle >30° with respect to the reference of the horizontal plane. In the analyzed groups, the number of saccades that did not meet one or more of these criteria was no more than the 15% of all saccades performed by each subject. The saccades were automatically identified through an *ad hoc* software and, subsequently, manually validated by two trained investigators. Saccades detection and parameter estimate algorithms were performed using Matlab software, version 7 (The MathWorks Inc., Natick, MA, USA).

### Saccade Definitions, Detection, and Analysis

In the anti-saccade task, the subjects are asked to execute an eye movement in the opposite direction from a visual stimulus onset. This movement implies the suppression of the reflexive tendency to look at the sudden onset stimulus (pro-saccade). Accordingly, we can give the following definitions: (a) a correct anti-saccade is the saccadic eye movement directed to the opposite direction in respect to visual target; (b) an erroneous pro-saccade is an uninhibited reflexive saccade toward the target; and (c) a corrective anti-saccade is an anti-saccade made after an initial erroneous pro-saccade.

For the analysis of the saccadic movements, we considered the following set of parameters: duration, peak and mean velocity, gain, and latency. The duration was calculated as the time interval between the start and the end of the movement; the peak velocity was the maximum eye velocity, in degrees of visual angle/second; gain was the ratio of the initial saccade amplitude to target distance, where we defined saccadic amplitude as the difference, in degrees of visual angle, between the eye position at the start and end of the saccade. The saccade latency was defined as the time delay between the target presentation and the saccade onset, and it was calculated also for erroneous pro-saccades and corrective anti-saccades. The latency of corrective anti-saccades includes the latency of an erroneous pro-saccade, its duration and the latency of the subsequent corrective anti-saccade. Moreover, we determined the intersaccadic latency between the end of the erroneous pro-saccade and the start of the corrective anti-saccade, when executed. Both measures (latency of corrective anti-saccades and intersaccadic latency) reflect anti-saccade programming. However, the intersaccadic latency is a more precise estimation of the anti-saccade programming. Indeed, if it is longer than the visual feedback of the error, the corrective anti-saccade is likely a movement generated after the visual error; if the intersaccadic latency is shorter than the visual feedback or the corresponding average latency of a correct anti-saccade, it is likely that the corrective anti-saccade is a pre-programmed movement generated before the visual feedback of the error.

Means and SDs of all saccadic parameters mentioned above were calculated for each task of each patient.

With respect to the gain, the mean value indicates the accuracy of the anti-saccades (closeness of the average amplitude to the actual target), while the reciprocal of the SD indicates the precision of the anti-saccades (compactness of grouping, or consistency, among amplitudes). Precisely, the greater the SD, the lower is the precision.

### Statistical Methods

Spearman rank order correlation coefficients were computed to measure the association among patient’s disability score (ICARS scale) and saccadic parameters. Either Kruskal–Wallis tests or Welch tests on ranked data were used to compare the effect of the group membership (controls, SCA2, and LOCA) on each saccadic parameter at 10 and 18 degrees and their variability among groups. Levene tests were used to assess homoscedasticity. A *post hoc* analysis was performed by Dunn multiple comparison test with Bonferroni adjustment. To verify the null hypothesis that the latencies of correctly executed anti-saccades were sampled from the same continuous distribution, Kolmogorov–Smirnov tests were set for pairwise comparisons of the latency distributions (controls vs LOCA, controls vs SCA2, LOCA vs SCA2).

The amplitude–duration and amplitude-peak velocity main sequences of the anti-saccades were considered. An exponential equation was used to fit peak velocity on amplitude, i.e., $V_{\text{peak}}=V_{\text{max}}*[1-e^{\text{Amplitude}/c}]$, where *V*_max_ is the asymptotic peak velocity and *c* is a constant. A linear fitting, i.e., Duration = *k* + *b* * Amplitude, where *k* is a constant and *b* is the slope of the fitted line, was adopted for the amplitude–duration relationship. The 95% prediction bounds around the healthy controls’ main sequences were also calculated. Percentage of direction errors with respect to the total number of anti-saccades trials and percentage of corrective anti-saccades with respect to the number of direction errors were compared among the three groups through χ^2^ tests (with two degrees of freedom). The Marascuilo procedure was applied to identify which proportions could be responsible for rejecting the null hypothesis of equal ratios. All tests were two-sided at the nominal significance level of 5%.

## Results

### Clinical and MRI Data

ICARS scores, MRI findings, and number of CAG repeats (for SCA2) of all patients are shown in Table 1. All SCA2 patients had been previously diagnosed by molecular analysis demonstrating abnormal CAG expansion (46). In these patients, MRI examination showed cerebellar atrophy extending to the middle cerebellar peduncle and midbrain, and the dorsal pons. Mild diffuse cortical atrophy was qualitatively observed in all SCA2 patients by two experienced neuroradiologists. In our SCA2 patients, while abnormality of the cerebral cortex was only qualitatively evaluated, but not quantitatively measured at the brain MRI, a cortical functional impairment could not be excluded given the common involvement in this disease. No basal ganglia changes were observed in these patients. No frank cognitive disabilities or dementia (MMSE score was ≥24 in all patients) were detected. Mean ICARS score in SCA2 population was 36.9/100. All LOCA subjects presented with pure cerebellar syndrome and MRI evidence of global cerebellar atrophy; no extra-cerebellar involvement was evident at MRI. Mean ICARS score in LOCA population was 33.8/100. No significant association between clinical disability assessment and anti-saccadic parameters were found in SCA2 and LOCA.

### Anti-Saccades

To better represent the results of anti-saccadic behavior, we are reporting separately the dynamic and metric outcome of the analysis, the latency, and the different measures of accuracy (Tables 2–4).

**Table 2:** Mean and SD values of dynamic and metric anti-saccade parameters.

		Duration (ms)	Peak velocity (deg/s)	Mean velocity (deg/s)	Gain [−]
CTR	10 deg	64.2 ± 19.3	339.2 ± 63.7	179.2 ± 35.7	0.98 ± 0.31
	18 deg	82.2 ± 11.9	433.3 ± 72.3	234.1 ± 38.0	0.96 ± 1.14

SCA2	10 deg	132.6 ± 58.9	202.2 ± 61.7	107.6 ± 30.4	1.08 ± 0.41
	18 deg	178.7 ± 40.4	191.6 ± 91.2	99.9 ± 45.1	0.83 ± 0.27

LOCA	10 deg	69.4 ± 12.1	379.6 ± 64.3	199.7 ± 33.6	1.22 ± 0.41
	18 deg	85.9 ± 19.4	471.2 ± 98.1	250.0 ± 53.9	0.94 ± 0.34

*Mean and SDs of duration, peak velocity, mean velocity, and gain of correctly executed anti-saccades estimated for each group (CTR, control group) separately at 10 and 18 deg. Statistical significance of comparisons among groups*.

*Duration: spinocerebellar ataxia type 2 (SCA2) vs CTR (10 and 18 deg, *p* < 0.001); SCA2 vs late onset cerebellar ataxia (LOCA) (18 deg, *p* < 0.001)*.

*Peak velocity: SCA2 vs CTR (10 and 18 deg, *p* < 0.001); SCA2 vs LOCA (10 and 18 deg, *p* < 0.001)*.

*Mean velocity: SCA2 vs CTR (10 and 18 deg, *p* < 0.001); SCA2 vs LOCA (10 and 18 deg, *p* < 0.001)*.

**Table 3:** SDs of anti-saccade parameters.

		Duration (ms)	Peak velocity (deg/s)	Mean velocity (deg/s)	Gain [−]	Latency (ms)
CTR	10 deg	58.6 (17.5)	70.1 (53.5)	41.4 (23.4)	0.20 (0.11)	41.6 (14.9)
	18 deg	80.7 (17.4)	78.9 (36.6)	48.7 (21.5)	0.14 (0.08)	31.6 (14.7)

SCA2	10 deg	117.8 (64.6)	53.3 (9.3)	24.6 (5.9)	0.47 (0.08)	35.4 (58.1)
	18 deg	185.6 (42.7)	39.6 (26.2)	18.1 (16.4)	0.20 (0.06)	64.8 (49.6)

LOCA	10 deg	68.5 (18.4)	106.4 (41.2)	51.7 (18.1)	0.57 (0.26)	71.3 (56.8)
	18 deg	83.3 (23.0)	93.5 (43.2)	56.0 (22.0)	0.18 (0.11)	62.8 (60.9)

*SDs (median and interquartile range) of duration, peak velocity, mean velocity, and gain of correctly executed anti-saccades estimated for each group (CTR, control group) separately at 10 and 18 deg. Statistical significance of comparisons among groups*.

*Latency: spinocerebellar ataxia type 2 (SCA2) vs CTR (18 deg, *p* < 0.05)*.

*Duration: SCA2 vs CTR (10 and 18 deg, *p* < 0.001); SCA2 vs late onset cerebellar ataxia (LOCA) (10 deg, *p* < 0.05; 18 deg, *p* < 0.001)*.

*Peak velocity: SCA2 vs CTR (18 deg, *p* < 0.001); LOCA vs controls (10 deg, *p* < 0.001); SCA2 vs LOCA (10 deg, *p* < 0.05; 18 deg, *p* < 0.001)*.

*Mean velocity: SCA2 vs LOCA (10 and 18 deg, *p* < 0.001); SCA2 vs controls (10 deg, *p* < 0.05; 18 deg, *p* < 0.001)*.

*Gain: SCA2 vs CTR (10 deg, *p* = 0.001; 18 deg, *p* < 0.05); LOCA vs controls (10 deg, *p* < 0.001)*.

**Table 4:** Mean and SD of latencies of correct anti-saccades, erroneous pro-saccades, corrective anti-saccades, and intersaccadic intervals.

		Anti-saccade latency (ms)	Erroneous pro-saccade latency (ms)	Corrective anti-saccade latency (ms)	Intersaccadic latency (ms)
CTR	10 deg	268.7 ± 30.8	184.2 ± 23.3	363.4 ± 43.6	127.5 ± 44.5
	18 deg	267.3 ± 28.3	204.9 ± 39.8	370.5 ± 67.1	93.2 ± 45.2

SCA2	10 deg	376.3 ± 83.8	260.4 ± 88.9	530.3 ± 53.1	252.6 ± 87.3
	18 deg	412.3 ± 133.8	264.3 ± 49.1	518.4 ± 100.1	219.4 ± 46.7

LOCA	10 deg	396.3 ± 109.3	213.8 ± 35.6	530.4 ± 115.4	240.4 ± 117.7
	18 deg	393.1 ± 61.34	236.9 ± 29.9	519.0 ± 121.1	223.4 ± 77.9

*Mean and SDs of latencies of correctly executed anti-saccades, erroneous pro-saccades, corrective anti-saccades, and intersaccadic intervals estimated for each group (CTR, control group) separately at 10 and 18 deg. Statistical significance of comparisons among groups*.

*Anti-saccades: spinocerebellar ataxia type 2 (SCA2) vs CTR (10 deg, *p* < 0.05; 18 deg, *p* < 0.001); late onset cerebellar ataxia (LOCA) vs controls (10 and 18 deg, *p* < 0.001)*.

*Erroneous pro-saccades: SCA2 vs CTR (18 deg, *p* < 0.05)*.

*Corrective anti-saccades: SCA2 vs CTR (10 deg, *p* < 0.001; 18 deg, *p* < 0.05); LOCA vs controls (10 and 18 deg, *p* < 0.05)*.

*Intersaccadic: SCA2 vs CTR (10 and 18 deg, *p* < 0.05); LOCA vs controls (10 deg, *p* < 0.05; 18 deg, *p* = 0.001)*.

#### Dynamic Parameters

**Figure 1:**
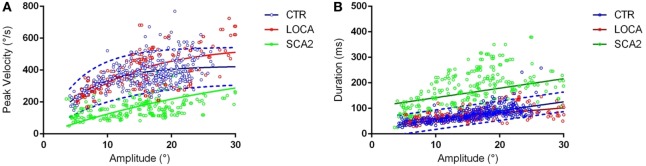
**(A)** Main sequences amplitude-peak velocity. Group-based different patterns of the relationship between peak velocity and amplitude were suggested by exploration of the amplitude-peak velocity main sequence. The thick lines denote the main sequence equations fitted over the data. The blue dashed lines represent the 95% prediction bound of the healthy controls. The scatter points of late onset cerebellar ataxia (LOCA) (red dots) and healthy controls (blue dots) overlapped almost everywhere, while the spinocerebellar ataxia type 2 (SCA2) data points (green dots) were outside the 95% prediction bounds. **(B)** Main sequences amplitude–duration. The SCA2 patients (green dots) presented also a different pattern in the amplitude–duration main sequence.

Late onset cerebellar ataxia patients showed slower anti-saccades than LOCA and controls (Table 2). In details: no differences were observed between controls and LOCA in average velocity and duration. SCA2 patients showed: (a) longer durations than controls (10 deg, *p* < 0.001; 18 deg, *p* < 0.001) and LOCA patients (18 deg, *p* < 0.001); (b) lower peak velocities than controls and LOCA (10 deg, *p* < 0.001; 18 deg, *p* < 0.001); and (c) lower mean velocity than controls and LOCA (10 deg, *p* < 0.001; 18 deg, *p* < 0.001) (Table 2). The SD of the anti-saccade dynamic parameters of healthy controls resulted lower than SCA2 (10 deg, *p* < 0.001; 18 deg, *p* < 0.001) and LOCA (10 deg, *p* = 0.009) (Table 3). The anti-saccades followed the main sequence relationships (peak velocity vs amplitude and duration vs amplitude, Figures 1A,B) in LOCA and control groups with no significant differences; SCA2 patients, indeed, did not follow the main sequence. In particular, SCA2 patients showed significantly lower peak velocity (*V*_max_ = 496.0 deg/s ± 73.3 deg/s, *C* = 34.6 ± 7.2) compared with healthy controls (*V*_max_ = 424.4 deg/s ± 8.2 deg/s, *C* = 7.0 ± 0.5) and LOCA (*V*_max_ = 551.4 deg/s ± 18.4 deg/s, *C* = 11.4 ± 0.8). In the amplitude–duration main sequence the duration in SCA2 patients (*k* = 104.8 ms ± 7.2 ms, *b* = 3.7 ± 0.3) resulted greater than LOCA (*k* = 41.1 ms ± 2.6 ms, *b* = 2.0 ± 0.1) and controls (*k* = 22.5 ms ± 2.5 ms, *b* = 3.4 ± 0.1).

#### Accuracy and Precision

The cerebellar patients showed overall good accuracy of anti-saccades, but worse precision than normal. Accuracy of correctly executed anti-saccades did not differ significantly among cerebellar patients (Table 2). Controls revealed significant higher precision in gain than LOCA (10 deg, *p* = 0.001) and SCA2 (10 deg, *p* < 0.001; 18 deg, *p* = 0.007) (Table 3).

#### Latency

**Figure 2:**
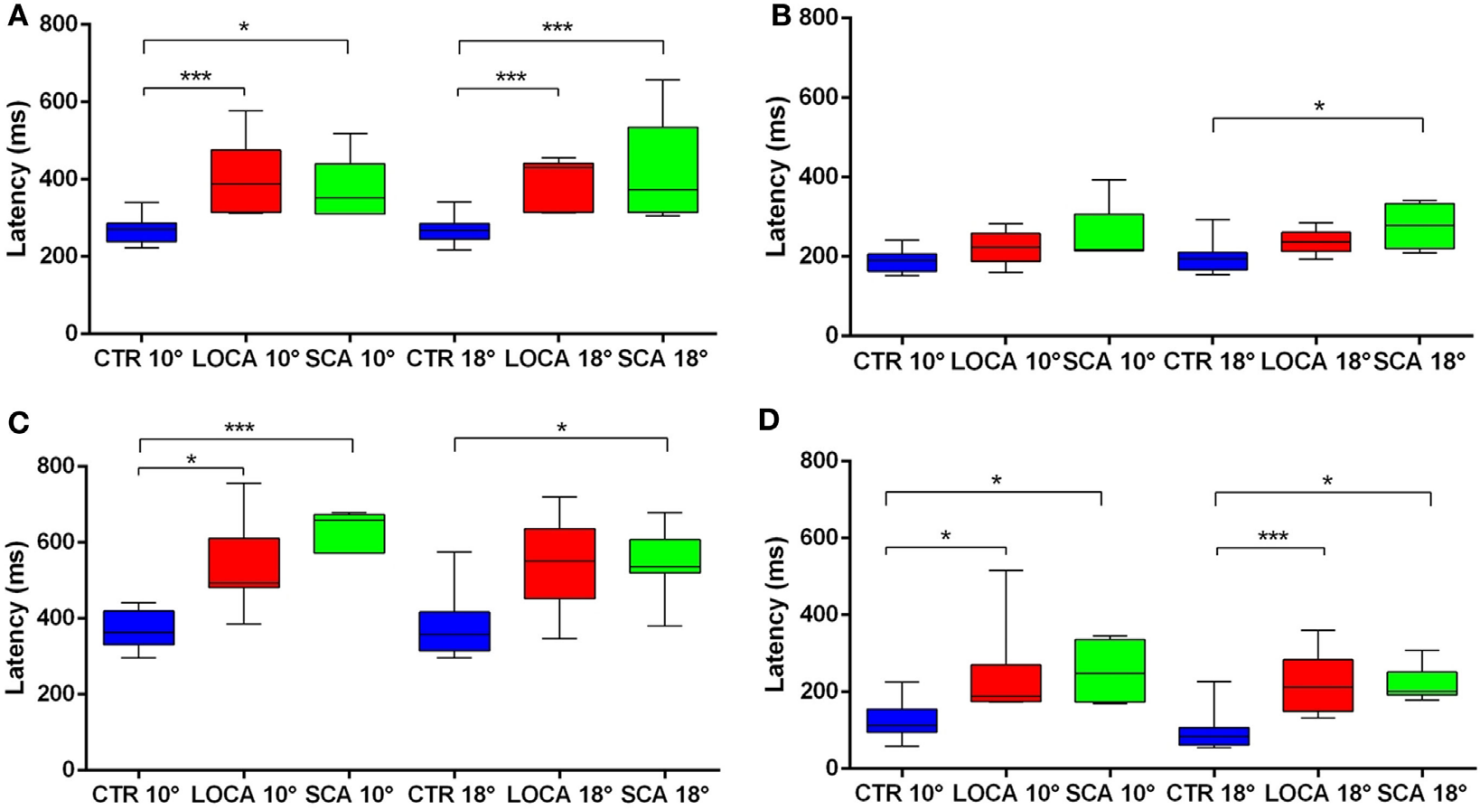
**(A–D)** Box plots of latencies by group and eccentricity. A star code is used for representing the extent of the significance levels of the differences in the pairwise comparisons: “***” if *p* ≤ 0.001 and “*” if *p* ≤ 0.05. **(A)** Box plots of latency of correctly executed anti-saccades. **(B)** Box plots of latency of erroneous pro-saccades. **(C)** Box plots of latency of corrective anti-saccades. **(D)** Box plots of inter-saccadic latency.

The cerebellar patients showed increased latency of all saccades (correct and corrective anti-saccades and pro-saccades) and longer intersaccadic intervals between erroneous pro-saccade and corrective anti-saccades (Table 4). The average latency of correctly executed anti-saccades was longer in SCA2 (10 deg, *p* = 0.003; 18 deg, *p* < 0.001) and LOCA (10 deg, *p* = 0.0012; 18 deg, *p* < 0.001) than controls. The latency of erroneous pro-saccades did not differ among groups at 10 deg. Latency of erroneous pro-saccade was significantly longer in SCA2 than controls at 18 deg (*p* = 0.015). The latency of corrective anti-saccades (with respect to the target onset) was significantly lower in the controls than LOCA (10 deg, *p* = 0.003; 18 deg, *p* = 0.005) and SCA2 patients (10 deg, *p* < 0.001; 18 deg, *p* = 0.003); no significant differences were observed between the two groups of patients. The intersaccadic latency was shorter in the controls than LOCA (10 deg, *p* = 0.004; 18 deg, *p* = 0.001) and SCA2 (10 deg, *p* = 0.011; 18 deg, *p* = 0.002), while no differences were observed between the two groups of patients (Table 4; Figures 2A–D).

**Figure 3:**
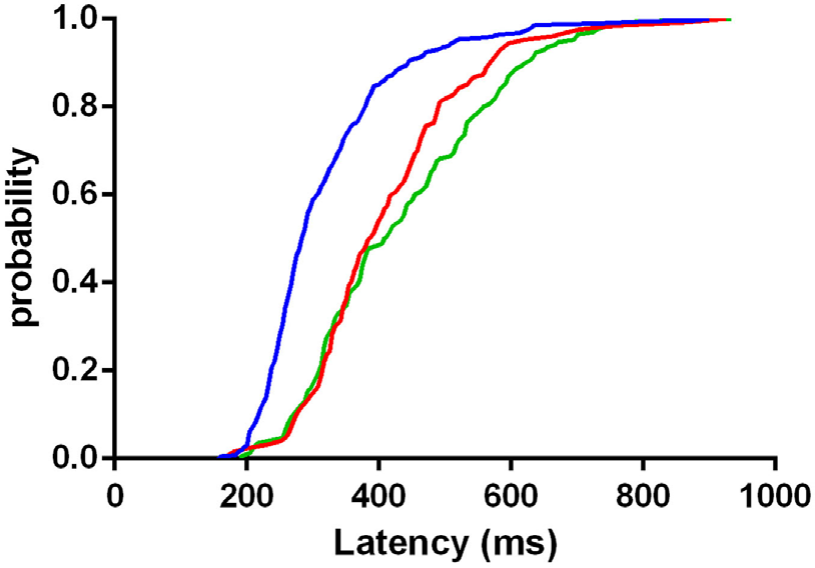
Cumulative distribution functions of latencies in correctly executed anti-saccades of controls (blue curve), late onset cerebellar ataxia (red curve), and spinocerebellar ataxia type 2 (green curve). The plot indicates that the mass of the distribution is more concentrated on the shortest values of latency in controls than in patients.

The distribution of latency of correctly executed anti-saccades was significantly less broadened in controls than both LOCA (*p* < 0.001) and SCA2 (*p* < 0.001). The distribution of latency did not differ significantly between LOCA and SCA2 (Figure 3).

#### Directional Error and Correction

**Figure 4:**
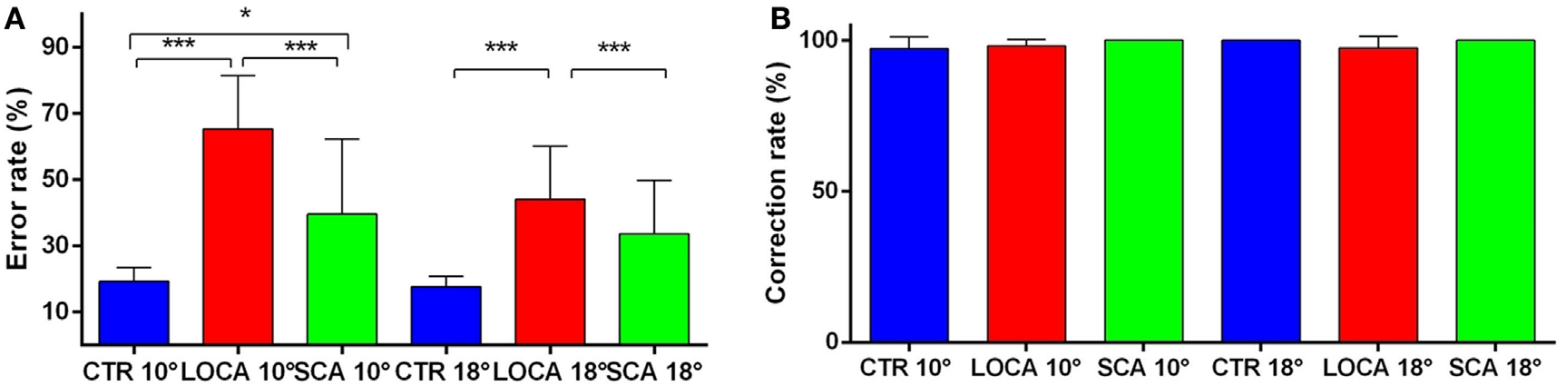
**(A)** Mean and SE of the percentage of errors in the anti-saccade task in each group at 10 and 18 deg. A star code is used for representing the extent of the significance levels of the differences in the pairwise comparisons: “***” if *p* ≤ 0.001 and “*” if *p* ≤ 0.05. **(B)** Mean and SE of the percentage of corrected anti-saccades with respect to the number of direction errors in each group at 10 and 18 deg.

The cerebellar patients made more directional error than normal, but corrected as frequently as controls. Indeed, the direction error rate (Figure 4A) was found significantly different among groups (10 deg, *p* < 0.001; 18 deg, *p* < 0.001). More precisely: (a) the error rate was higher in SCA2 (39.6%) than controls (19.3%) (percentage difference, 20.3; critical value, 10.61); (b) in LOCA (65.4%) than controls (percentage difference, 46.1; critical value, 10.36); and (c) the percentage of errors in LOCA was significantly higher than in SCA2 (percentage difference, 25.82; critical value, 13.02). The percentage of corrections among healthy controls (10 deg, 97.2%; 18 deg, 100%), SCA2 (10 deg, 100%; 18 deg, 100%), and LOCA (10 deg, 98%; 18 deg, 97.4%) did not result in significant differences at both target eccentricities (Figure 4B).

## Discussion

The observation of executive functions impairment in cerebellar patients is one evidence in favor of a cerebellar role in cognitive activity (25–27, 30–32, 47, 48). Nevertheless, the role of the cerebellum in executive functions control has been poorly differentiated from that of cerebral cortical areas, in particular the frontal cortex.

Therefore, in this study, in order to better understand the role of the cerebellum in controlling the anti-saccades and ultimately its role in controlling executive functions, we compared the anti-saccadic behavior of two pathologically different groups of cerebellar neurodegenerative diseases: SCA2 with and LOCA without involvement of the frontal cortex. We found increased latency of anti-saccades in both groups of cerebellar patients with respect to controls. The cerebellar patients made also more directional errors, but their errors were corrected as frequently as controls. No significant differences were found in patients with and without cortical involvement. Other saccadic dynamic parameters of anti-saccades retained the same characteristics of pro-saccades [see Federighi et al. (49) for a description of pro-saccades in these patients]. Indeed, according to impairment of the brainstem burst generator and OMV, SCA2 showed slower anti-saccades, and LOCA faster anti-saccades (50). Furthermore, metric parameters, such as velocity, were affected by a greater variability in both cerebellar patients than controls.

The anti-saccades are an effective tool to study executive functions, since their underlying neural network has been relatively well clarified. This often allows the specific identification of which step, and therefore related cerebral area, might be impaired in case of defective performance.

In particular, the frontal eye field (FEF) is supposed to plan the correct anti-saccade while, along with the dorso-lateral pre-frontal cortex (51–53), it evaluates the appropriateness of the saccade response with respect to the specific context, avoiding inappropriate responses (54–59). The pre-supplementary motor area (pre-SMA) and supplementary eye field (SEF) (60–63) predispose the oculomotor system for the anti-saccade response over the prevalent pro-saccade reaction. The anterior cingulate cortex is also supposed to contribute to the anti-saccade planning by monitoring the resolution of conflict between reflexive and anti-saccades (55, 64). In the posterior parietal cortex, the parietal eye field (PEF) is involved in computing the spatial location of the stimulus (65–67) and thus the vector of the required anti-saccade in spatial coordinates (68, 69). The basal ganglia may participate in inhibiting the pro-saccade and facilitate the anti-saccade (20, 70). Finally, the superior colliculus releases the fixation and makes the ultimate decision on the pro-saccade/anti-saccade execution. As expected, abnormalities of anti-saccades are extensively reported in parietal and frontal lobe dysfunctions. More specifically, human lesions and transcranial magnetic stimulation (TMS) interference in FEF have been associated with increased error rate, lesions of SEF, and pre-SMA with longer latency; while extensive lesions of frontal lobe have caused inability to perform the anti-saccadic task (15, 17, 61, 71, 72). Moreover, PEF damage and TMS application have been related to hypometric and less precise anti-saccades with longer latency (51, 73, 74).

The cerebellum is also supposed to participate to this complex network by controlling voluntary eye movements through its connections with the frontal-parietal cortex (75, 76).

Indeed, anti-saccades increased error rate and prolonged latencies have been found in patients with Cerebrotendineous Xanthomatosis (24), Friedreich ataxia (25), Spinocerebellar Ataxia type 1 (26), SCA2 (27), focal cerebellar lesions (28), and degenerative cerebellar diseases (29–31). A significant correlation between high-error rates of anti-saccades and loss of gray matter volume in the posterolateral cerebellum has also been reported in neurodegenerative cerebellar diseases (77). This cerebellar region has been associated with complex motor and cognitive functions (78) and is also activated in concert with the pre-frontal cortex, *via* projections to the thalamus and SMA, during error processing and post error processing in stop signal and reversal tasks (79, 80).

However, most patients with cerebellar degenerative diseases, who have been enrolled in these studies, presented atrophy that was not confined to the cerebellum, but involved also cortical areas. Therefore, in these studies, while a role of the cerebellum can be strongly suspected, its contribution cannot be isolated.

To our knowledge, this is the first study in which two large groups of cerebellar patients with and without cortical involvement are compared in order to identify the role of the cerebellum in the anti-saccade execution. As anticipated above, according with previous studies, our results demonstrated that all cerebellar patients presented an increased rate of unwanted pro-saccades in the anti-saccadic task. Since no significant differences were found between SCA2 and LOCA, this observation suggests a direct control of the cerebellum on the frontal network in suppressing an unwanted reflexive movement when a competing voluntary movement is desired (37, 81–85). Thus, the cerebellum, which is reciprocally interconnected with frontal areas inhibiting reflexive movements (FEF and DLPFC), could play a central role in the decisional process of voluntary motor control.

Also, in agreement with previous studies, we found that cerebellar patients had increased and more variable anti-saccadic latency than the normal population (14, 22, 25, 27, 77). Previously, it has been demonstrated that the latency of anti-saccades is usually more variable (trial by trial variability) and longer than that of visually guided saccades (12, 56). An influential model explaining the response time distribution in a simple decisional process such as pro-saccades vs anti-saccade, is the linear approach to threshold with ergodic rate model. This is a race-to-threshold model in which two different options compete for the final decision. According to rise-to-threshold models of anti-saccades (61, 86, 87), we suggest that longer and variable latencies might reflect the unpredictable timing for the accumulation of information concerning the execution of a correct anti-saccade (88), or might result from noisy accumulation of information during the decisional process (89, 90). Since a cerebellar dysfunction alone seems to be sufficient to delay the latency of a wanted movement (an anti-saccade in this case), a specific role of the cerebellum in controlling the onset of a voluntary movement is likely. The cerebellum, thus, through its connections, might accelerate the information accumulation or reduce the noise in pre-frontal areas like the SEF and pre-SMA, and/or in the parietal areas as the PEF, which are involved in determining the anti-saccade onset.

As novel findings, we showed that cerebellar patients corrected their errors as frequently as normal, but the corrective anti-saccades occurred at a longer latency than normal.

In a previous study, we had already observed that, while the rate of anti-saccade errors increased with subcortical impairment (namely cerebellum and basal ganglia dysfunction), the rate of anti-saccade correction decreased only when also a cerebral cortical involvement and cognitive impairment was present (38). In this framework, the cerebellum would intervene in facilitating the suppression of unwanted reflexive movements (pro-saccades), but in case of errors, their correction would be mostly a cortical property. Also in this study, the impairment of the cerebellum was associated with increased directional error rate. Conversely, the well-maintained correction rate in our subjects might indicate a relative preservation of the cortical areas (as otherwise indicated by the absence of severe cognitive impairment in all our patients including the SCA2 patients). This would suggest an intrinsic monitoring of the failure/correction rate in the pre-frontal areas during goal-directed movements; on this rate of correction, subcortical areas such as the cerebellum would have little influence.

It is noteworthy that the corrective anti-saccades in the cerebellar patients occurred at longer intersaccadic intervals than normal. In normal subjects, the intersaccadic interval between erroneous pro-saccade and corrective anti-saccade was quite short for allowing a visual feedback and clearly shorter than the average latency of a correct anti-saccade. This indicates that the corrective anti-saccade was likely pre-planned and not generated after the visual feedback of the error. Also in cerebellar patients the error-correction intersaccadic interval was shorter than the correct anti-saccade latency, thus suggesting that the corrective anti-saccades were possibly pre-planned in their case as well.

Overall, we did not find significant differences between SCA2 and LOCA in anti-saccade behavior. This is perhaps surprising even considering a relative sparing of the cortical areas in our SCA2 patients, because we would have nevertheless expected a cumulative effect of their neurodegeration (cortex plus cerebellum) to result in a worse performance. The lack of significative difference might instead suggest that the cerebellum plays a crucial, bottleneck role in executive control of anti-saccades. Moreover, it might indicate that the cortical involvement (particularly the frontal lobe impairment) might be compensated by other areas, including those spared in the cerebellum. Supporting this hypothesis could be the finding of relatively spared dentate nuclei in the same SCA2 patients (91–93). This also suggests that the cerebellum has more than an ancillary supporting role in executive control, being able to compensate up to a certain measure a cortical failure.

In conclusion, our study sustains and reinforces the hypothesis that the cerebellum contributes actively to the control of executive functions. The cerebellum would participate in a functional network connecting the pre-frontal, frontal, and parietal cortex that facilitates the planning of voluntary movements while suppressing perturbing automatic responses. However, not all cortical computations might be under the same cerebellar control. Some cortical properties, such as the failure/correction rate, might be less easily influenced by the cerebellum.

## Ethics Statement

All procedures performed in studies involving human participants were in accordance with the ethical standards of Local Ethic Committee: Comitato Etico Locale Azienda Ospedaliera Universitaria Senese and with the 1964 Helsinki declaration and its later amendments or comparable ethical standards. The protocol was approved by the local Ethical Committee (EVAlab protocol CEL no. 48/2010). Patients gave their written consent.

## Author Contributions

EP performed and analyzed saccades, interpreted results, and wrote the paper; PP performed statistical analysis, interpreted results, and wrote tables and figures; FR collected clinical findings, performed, and analyzed saccades, interpreted results; PF implemented saccade analysis and interpreted results; VS wrote “Materials and Methods” paragraph and gave her contribution in saccade analysis and figure plotting; GT analyzed saccades and collected patient’s data; MD and AF collected patients and defined the clinical and genetic diagnosis; AR evaluated patients, designed the study, interpreted, and wrote the paper.

## Conflict of Interest Statement

The authors declare that the research was conducted in the absence of any commercial or financial relationships that could be construed as a potential conflict of interest.

## Abbreviations

ACC, anterior cingulate cortex; BG, Basal Ganglia; DLPC, dorso-lateral pre-frontal cortex; FEF, frontal eye field; ICARS, International Cooperative Ataxia Rating Scale; LOCA, late-onset cerebellar ataxia; OMV, oculomotor vermis; PEF, parietal eye field; PPC, posterior parietal cortex; SCA2, spinocerebellar ataxia type 2; SEF, supplementary eye field; SMA, supplementary motor area; TMS, transcranial magnetic stimulation.

## References

[1] Hopp JJ Fuchs AF. The characteristics and neuronal substrate of saccadic eye movement plasticity. Prog Neurobiol (2004) 72:27–53. 10.1016/j.pneurobio.2003.12.002 15019175

[2] Catz N Dicke PW Thier P. Cerebellar complex spike firing is suitable to induce as well as to stabilize motor learning. Curr Biol (2005) 15:2179–89. 10.1016/j.cub.2005.11.037 16360681

[3] Chen-Harris H Joiner WM Ethier V Zee DS Shadmehr R. Adaptive control of saccades via internal feedback. J Neurosci (2008) 28:2804–13. 10.1523/JNEUROSCI.5300-07.2008 18337410 PMC2733833

[4] Kojima Y Soetedjo R Fuchs AF. Behavior of the oculomotor vermis for five different types of saccade. J Neurophysiol (2010) 104:3667–76. 10.1152/jn.00558.2010 20962069 PMC3007629

[5] Allen G Courchesne E The cerebellum and non-motor function: clinical implications. Mol Psychiatry (1998) 3:207–10. 10.1038/sj.mp.4000395 9672891

[6] Dum RP Strick PL. An unfolded map of the cerebellar dentate nucleus and its projections to the cerebral cortex. J Neurophysiol (2003) 89:634–9. 10.1152/jn.00626.2002 12522208

[7] Manto M Lorivel T Cognitive repercussions of hereditary cerebellar disorders. Cortex (2011) 47:81–100. 10.1016/j.cortex.2009.04.012 19539904

[8] Hocking DR Corben LA Fielding J Cremer PD Millist L White OB Saccade reprogramming in Friedreich ataxia reveals impairments in the cognitive control of saccadic eye movement. Brain Cogn (2014) 87:161–7. 10.1016/j.bandc.2014.03.018 24752035

[9] Gao JH Parsons LM Bower JM Xiong J Li J Fox PT. Cerebellum implicated in sensory acquisition and discrimination rather than motor control. Science (1996) 272:545–7. 10.1126/science.272.5261.545 8614803

[10] Blakemore SJ Frith CD Wolpert DM. Spatio-temporal prediction modulates the perception of self-produced stimuli. J Cogn Neurosci (1999) 11:551–9. 10.1162/089892999563607 10511643

[11] Blakemore SJ Wolpert DM Frith CD. The cerebellum contributes to somatosensory cortical activity during self-produced tactile stimulation. Neuroimage (1999) 10:448–59. 10.1006/nimg.1999.0478 10493902

[12] Peterburs J Desmond JE. The role of the human cerebellum in performance monitoring. Curr Opin Neurobiol (2016) 40:38–44. 10.1016/j.conb.2016.06.011 27372055 PMC5056810

[13] Buckner RL. The cerebellum and cognitive function: 25 years of insight from anatomy and neuroimaging. Neuron (2013) 80:807–15. 10.1016/j.neuron.2013.10.044 24183029

[14] Hallett PE Primary and secondary saccades to goals defined by instructions. Vision Res (1978) 18:1279–96. 10.1016/0042-6989(78)90218-3 726270

[15] Terao Y Fukuda H Ugawa Y Hikosaka O Hanajima R Furubayashi T Visualization of the information flow through human oculomotor cortical regions by transcranial magnetic stimulation. J Neurophysiol (1998) 80:936–46. 10.1152/jn.1998.80.2.936 9705480

[16] Middleton FA Strick PL. Cerebellar projections to the prefrontal cortex of the primate. J Neurosci (2001) 21:700–12. 10.1523/JNEUROSCI.21-02-00700.2001 11160449 PMC6763818

[17] Curtis CE D’Esposito M. Success and failure suppressing reflexive behavior. J Cogn Neurosci (2003) 15:409–18. 10.1162/089892903321593126 12729492

[18] Ford KA Goltz HC Brown MR Everling S. Neural processes associated with antisaccade task performance investigated with event-related FMRI. J Neurophysiol (2005) 94:429–40. 10.1152/jn.00471.2004 15728770

[19] McDowell JE Kissler JM Berg P Dyckman KA Gao Y Rockstroh B Electroencephalography/magnetoencephalography study of cortical activities preceding prosaccades and antisaccades. Neuroreport (2005) 16:663–8. 10.1097/00001756-200505120-00002 15858402

[20] Ettinger U Ffytche DH Kumari V Kathmann N Reuter B Zelaya F Decomposing the neural correlates of antisaccade eye movements using event-related FMRI. Cereb Cortex (2008) 18:1148–59. 10.1093/cercor/bhm147 17728263

[21] de Weijer AD Mandl RC Sommer IE Vink M Kahn RS Neggers SF. Human fronto-tectal and fronto-striatal-tectal pathways activate differently during anti-saccades. Front Hum Neurosci (2010) 4:41. 10.3389/fnhum.2010.00041 20631846 PMC2903195

[22] Prevosto V Graf W Ugolini G. Cerebellar inputs to intraparietal cortex areas LIP and MIP: functional frameworks for adaptive control of eye movements, reaching, and arm/eye/head movement coordination. Cereb Cortex (2010) 20:214–28. 10.1093/cercor/bhp091 19465740 PMC2860711

[23] Ramnani N. Frontal lobe and posterior parietal contributions to the cortico-cerebellar system. Cerebellum (2012) 11:366–83. 10.1007/s12311-011-0272-3 21671065

[24] Rosini F Pretegiani E Mignarri A Optican LM Serchi V De Stefano N The role of dentate nuclei in human oculomotor control: insights from cerebrotendinous xanthomatosis. J Physiol (2017) 595:3607–20. 10.1113/JP273670 28168705 PMC5451708

[25] Fielding J Corben L Cremer P Millist L White O Delatycki M. Disruption to higher order processes in Friedreich ataxia. Neuropsychologia (2010) 48:235–42. 10.1016/j.neuropsychologia.2009.09.009 19766130

[26] Rivaud-Pechoux S Dürr A Gaymard B Cancel G Ploner CJ Agid Y Eye movement abnormalities correlate with genotype in autosomal dominant cerebellar ataxia type I. Ann Neurol (1998) 43:297–302. 10.1002/ana.410430306 9506545

[27] Rodríguez-Labrada R Velázquez-Pérez L Aguilera-Rodríguez R Seifried-Oberschmidt C Peña-Acosta A Canales-Ochoa N Executive deficit in spinocerebellar ataxia type 2 is related to expanded CAG repeats: evidence from antisaccadic eye movements. Brain Cogn (2014) 91:28–34. 10.1016/j.bandc.2014.07.007 25189938

[28] Brunamonti E Chiricozzi FR Clausi S Olivito G Giusti MA Molinari M Cerebellar damage impairs executive control and monitoring of movement generation. PLoS One (2014) 9:e85997. 10.1371/journal.pone.0085997 24465830 PMC3895022

[29] King SA Schneider RM Serra A Leigh RJ. Critical role of cerebellar fastigial nucleus in programming sequences of saccades. Ann N Y Acad Sci (2011) 1233:155–61. 10.1111/j.1749-6632.2011.06119.x 21950988 PMC3187558

[30] Filippopulos F Eggert T Straube A. Deficits of cortical oculomotor mechanisms in cerebellar atrophy patients. Exp Brain Res (2013) 224:541–50. 10.1007/s00221-012-3332-0 23161158

[31] Jamadar SD Johnson BP Clough M Egan GF Fielding J. Behavioral and neural plasticity of ocular motor control: changes in performance and fMRI activity following antisaccade training. Front Hum Neurosci (2015) 9:653. 10.3389/fnhum.2015.00653 26733841 PMC4683540

[32] Robinson DA Oculomotor control signals. In: Lennerstrand F Bach-y-Rita P, editors. Basic Mechanisms of Ocular Motility and Their Clinical Implications. Oxford: Pergamon Press (1975). p. 337–74.

[33] Dieterich M Bucher SF Seelos KC Brandt T. Cerebellar activation during optokinetic stimulation and saccades. Neurology (2000) 54:148–55. 10.1212/WNL.54.1.148 10636141

[34] Robinson FR Fuchs AF. The role of the cerebellum in voluntary eye movements. Annu Rev Neurosci (2001) 24:981–1004. 10.1146/annurev.neuro.24.1.981 11520925

[35] Nitschke MF Binkofski F Buccino G Posse S Erdmann C Kömpf D Activation of cerebellar hemispheres in spatial memorization of saccadic eye movements: an fMRI study. Hum Brain Mapp (2004) 22:155–64. 10.1002/hbm.20025 15108303 PMC6871891

[36] Nitschke MF Arp T Stavrou G Erdmann C Heide W The cerebellum in the cerebro-cerebellar network for the control of eye and hand movements—an fMRI study. Prog Brain Res (2005) 148:151–64. 10.1016/S0079-6123(04)48013-3 15661188

[37] Strick PL Dum RP Fiez JA. Cerebellum and nonmotor function. Annu Rev Neurosci (2009) 32:413–34. 10.1146/annurev.neuro.31.060407.125606 19555291

[38] Pretegiani E Rosini F Federico A Optican LM Rufa A Eye movements in genetic parkinsonisms affecting the α-synuclein, PARK9, and manganese network. Clin Neurophysiol (2017) 128:2450–3. 10.1016/j.clinph.2017.09.107 29096219

[39] Kerber KA Jen JC Perlman S Baloh RW. Late-onset pure cerebellar ataxia: differentiating those with and without identifiable mutations. J Neurol Sci (2005) 238:41–5. 10.1016/j.jns.2005.06.006 16109427

[40] Magaña JJ Velázquez-Pérez L Cisneros B. Spinocerebellar ataxia type 2: clinical presentation, molecular mechanisms, and therapeutic perspectives. Mol Neurobiol (2013) 47:90–104. 10.1007/s12035-012-8348-8 22996397

[41] Lastres-Becker I Rüb U Auburger G Spinocerebellar ataxia 2 (SCA2). Cerebellum (2008) 7:115–24. 10.1007/s12311-008-0019-y 18418684

[42] Rüb U Schöls L Paulson H Auburger G Kermer P Jen JC Clinical features, neurogenetics and neuropathology of the polyglutamine spinocerebellar ataxias type 1, 2, 3, 6 and 7. Prog Neurobiol (2013) 104:38–66. 10.1016/j.pneurobio.2013.01.001 23438480

[43] Trouillas P Takayanagi T Hallett M Currier RD Subramony SH Welles K International cooperative ataxia rating scale for pharmacological assessment of the cerebellar syndrome. The ataxia neuropharmacology committee of the world federation of neurology. J Neurol Sci (1997) 145:205–11. 10.1016/S0022-510X(96)00231-6 9094050

[44] Leigh RJ Zee DS The Neurology of Eye Movements. 5th ed New York: Oxford University Press (2015).

[45] Inchingolo P Spanio M On the identification and analysis of saccadic eye movements—a quantitative study of the processing procedures. IEEE Trans Biomed Eng (1985) 32:683–95. 10.1109/TBME.1985.325586 4054932

[46] Pareyson D Gellera C Castellotti B Antonelli A Riggio MC Mazzucchelli F Clinical and molecular studies of 73 Italian families with autosomal dominant cerebellar ataxia type I: SCA1 and SCA2 are the most common genotypes. J Neurol (1999) 246:389–93. 10.1007/s004150050369 10399872

[47] Hübner J Sprenger A Klein C Hagenah J Rambold H Zühlke C Eye movement abnormalities in spinocerebellar ataxia type 17 (SCA17). Neurology (2007) 69:1160–8. 10.1212/01.wnl.0000276958.91986.89 17846415

[48] Shelton AL Cornish K Kraan C Georgiou-Karistianis N Metcalfe SA Bradshaw JL Exploring inhibitory deficits in female premutation carriers of fragile X syndrome: through eye movements. Brain Cogn (2014) 85:201–8. 10.1016/j.bandc.2013.12.006 24424424

[49] Federighi P Cevenini G Dotti MT Rosini F Pretegiani E Federico A Differences in saccade dynamics between spinocerebellar ataxia 2 and late-onset cerebellar ataxias. Brain (2011) 134:879–91. 10.1093/brain/awr009 21354979

[50] Krappmann P Everling S Flohr H. Accuracy of visually and memory-guided antisaccades in man. Vision Res (1998) 38:2979–85. 10.1016/S0042-6989(98)00101-1 9797993

[51] Gaymard B Lynch J Ploner CJ Condy C Rivaud-Péchoux S. The parieto-collicular pathway: anatomical location and contribution to saccade generation. Eur J Neurosci (2003) 17:1518–26. 10.1046/j.1460-9568.2003.02570.x 12713655

[52] Pierrot-Deseilligny C Müri RM Ploner CJ Gaymard B Demeret S Rivaud-Pechoux S. Decisional role of the dorsolateral prefrontal cortex in ocular motor behaviour. Brain (2003) 126:1460–73. 10.1093/brain/awg148 12764065

[53] Pierrot-Deseilligny C Milea D Müri RM. Eye movement control by the cerebral cortex. Curr Opin Neurol (2004) 17:17–25. 10.1097/00019052-200402000-00005 15090873

[54] Bruce CJ Goldberg ME. Primate frontal eye fields. I. Single neurons discharging before saccades. J Neurophysiol (1985) 53:603–35. 10.1152/jn.1985.53.3.603 3981231

[55] Everling S Johnston K. Control of the superior colliculus by the lateral prefrontal cortex. Philos Trans R Soc Lond B Biol Sci (2013) 368:20130068. 10.1098/rstb.2013.0068 24018729 PMC3758210

[56] Everling S Munoz DP Neuronal correlates for preparatory set associated with prosaccades and anti-saccades in the primate frontal eye field. J Neurosci (2000) 20:387–400. 10.1523/JNEUROSCI.20-01-00387.2000 10627615 PMC6774131

[57] Guitton D Buchtel HA Douglas RM. Frontal lobe lesions in man cause difficulties in suppressing reflexive glances and in generating goal-directed saccades. Exp Brain Res (1985) 58:455–72. 10.1007/BF00235863 4007089

[58] Connolly JD Goodale MA Menon RS Munoz DP. Human fMRI evidence for the neural correlates of preparatory set. Nat Neurosci (2002) 5:1345–52. 10.1038/nn969 12411958

[59] Jamadar SD Fielding J Egan GF. Quantitative meta-analysis of fMRI and PET studies reveals consistent activation in fronto-striatal-parietal regions and cerebellum during antisaccades and prosaccades. Front Psychol (2013) 4:749. 10.3389/fpsyg.2013.00749 24137150 PMC3797465

[60] Schlag-Rey M Amador N Sanchez H Schlag J. Antisaccade performance predicted by neuronal activity in the supplementary eye field. Nature (1997) 390:398–401. 10.1038/37114 9389478

[61] Stuphorn V Schall JD. Neuronal control and monitoring of initiation of movements. Muscle Nerve (2002) 26:326–39. 10.1002/mus.10158 12210361

[62] Amador N Schlag-Rey M Schlag J. Primate antisaccade. II. Supplementary eye field neuronal activity predicts correct performance. J Neurophysiol (2004) 91:1672–89. 10.1152/jn.00138.2003 14645374

[63] Carpenter RH Contrast, probability, and saccadic latency; evidence for independence of detection and decision. Curr Biol (2004) 14:1576–80. 10.1016/j.cub.2004.08.058 15341745

[64] Botvinick MM Cohen JD Carter CS. Conflict monitoring and anterior cingulate cortex: an update. Trends Cogn Sci (2004) 8:539–46. 10.1016/j.tics.2004.10.003 15556023

[65] Duhamel JR Goldberg ME Fitzgibbon EJ Sirigu A Grafman J. Saccadic dysmetria in a patient with a right frontoparietal lesion. The importance of corollary discharge for accurate spatial behaviour. Brain (1992) 115:1387–402. 10.1093/brain/115.5.1387 1422794

[66] Colby CL Goldberg ME. Space and attention in parietal cortex. Annu Rev Neurosci (1999) 22:319–49. 10.1146/annurev.neuro.22.1.319 10202542

[67] Gottlieb J Goldberg ME. Activity of neurons in the lateral intraparietal area of the monkey during an antisaccade task. Nat Neurosci (1999) 2:906–12. 10.1038/13209 10491612

[68] Zhang M Barash S. Neuronal switching of sensorimotor transformations for antisaccades. Nature (2000) 408:971–5. 10.1038/35050097 11140683

[69] Zhang M Barash S. Persistent LIP activity in memory antisaccades: working memory for a sensorimotor transformation. J Neurophysiol (2004) 91:1424–41. 10.1152/jn.00504.2003 14523076

[70] Kim HF Hikosaka O. Parallel basal ganglia circuits for voluntary and automatic behaviour to reach rewards. Brain (2015) 138:1776–800. 10.1093/brain/awv134 25981958 PMC4492412

[71] Schlag-Rey M Schlag J Dassonville P. How the frontal eye field can impose a saccade goal on superior colliculus neurons. J Neurophysiol (1992) 67:1003–5. 10.1152/jn.1992.67.4.1003 1588383

[72] Boxer AL Garbutt S Rankin KP Hellmuth J Neuhaus J Miller BL Medial versus lateral frontal lobe contributions to voluntary saccade control as revealed by the study of patients with frontal lobe degeneration. J Neurosci (2006) 26:6354–63. 10.1523/JNEUROSCI.0549-06.2006 16763044 PMC2551317

[73] Gottlieb J Kusunoki M Goldberg ME. Simultaneous representation of saccade targets and visual onsets in monkey lateral intraparietal area. Cereb Cortex (2005) 15:1198–206. 10.1093/cercor/bhi002 15616137 PMC2377182

[74] Andersen RA Brotchie PR Mazzoni P. Evidence for the lateral intraparietal area as the parietal eye field. Curr Opin Neurobiol (1992) 2:840–6. 10.1016/0959-4388(92)90143-9 1477549

[75] Middleton FA Strick PL. Anatomical evidence for cerebellar and basal ganglia involvement in higher cognitive function. Science (1994) 266:458–61. 10.1126/science.7939688 7939688

[76] Stoodley CJ. The cerebellum and cognition: evidence from functional imaging studies. Cerebellum (2012) 11:352–65. 10.1007/s12311-011-0260-7 21373864

[77] Peterburs J Thürling M Rustemeier M Göricke S Suchan B Timmann D A cerebellar role in performance monitoring—evidence from EEG and voxel-based morphometry in patients with cerebellar degenerative disease. Neuropsychologia (2015) 68:139–47. 10.1016/j.neuropsychologia.2015.01.017 25592368

[78] Stoodley CJ Schmahmann JD. Functional topography in the human cerebellum: a meta-analysis of neuroimaging studies. Neuroimage (2009) 44:489–501. 10.1016/j.neuroimage.2008.08.039 18835452

[79] Ide JS Li CS. A cerebellar thalamic cortical circuit for error-related cognitive control. Neuroimage (2011) 54:455–64. 10.1016/j.neuroimage.2010.07.042 20656038 PMC2962720

[80] Von der Gablentz J Tempelmann C Münte TF Heldmann M. Performance monitoring and behavioral adaptation during task switching: an fMRI study. Neuroscience (2015) 285:227–35. 10.1016/j.neuroscience.2014.11.024 25446349

[81] Okuda B. Cerebello-thalamo-cerebral projection from the dentate nucleus onto the frontal eye field in the cat. Acta Physiol Scand (1994) 151:1–6. 10.1111/j.1748-1716.1994.tb09715.x 8048330

[82] Luna B Thulborn KR Munoz DP Merriam EP Garver KE Minshew NJ Maturation of widely distributed brain function subserves cognitive development. Neuroimage (2001) 13:786–93. 10.1006/nimg.2000.0743 11304075

[83] Nagao S. Pontine nuclei-mediated cerebello-cerebral interactions and its functional role. Cerebellum (2004) 3:11–5. 10.1080/14734220310012181 15072262

[84] Prevosto V Sommer MA. Cognitive control of movement via the cerebellar-recipient thalamus. Front Syst Neurosci (2013) 7:56. 10.3389/fnsys.2013.00056 24101896 PMC3787245

[85] Hashimoto M Takahara D Hirata Y Inoue K Miyachi S Nambu A Motor and non-motor projections from the cerebellum to rostrocaudally distinct sectors of the dorsal premotor cortex in macaques. Eur J Neurosci (2010) 31:1402–13. 10.1111/j.1460-9568.2010.07151.x 20384784

[86] Kristjánsson A Chen Y Nakayama K. Less attention is more in the preparation of antisaccades, but not prosaccades. Nat Neurosci (2001) 4:1037–42. 10.1038/nn723 11547337

[87] Munoz DP Everling S Look away: the anti-saccade task and the voluntary control of eye movement. Nat Rev Neurosci (2004) 5:218–28. 10.1038/nrn1345 14976521

[88] Noorani I Carpenter RH. Antisaccades as decisions: LATER model predicts latency distributions and error responses. Eur J Neurosci (2013) 37:330–8. 10.1111/ejn.12025 23121177

[89] Cutsuridis V Smyrnis N Evdokimidis I Perantonis S. A neural model of decision-making by the superior colicullus in an antisaccade task. Neural Netw (2007) 20:690–704. 10.1016/j.neunet.2007.01.004 17446043

[90] Cutsuridis V Kumari V Ettinger U. Antisaccade performance in schizophrenia: a neural model of decision making in the superior colliculus. Front Neurosci (2014) 8:13. 10.3389/fnins.2014.00013 24574953 PMC3920187

[91] Mascalchi M Diciotti S Giannelli M Ginestroni A Soricelli A Nicolai E Progression of brain atrophy in spinocerebellar ataxia type 2: a longitudinal tensor-based morphometry study. PLoS One (2014) 9:e89410. 10.1371/journal.pone.0089410 24586758 PMC3934889

[92] Della Nave R Ginestroni A Tessa C Salvatore E De Grandis D Plasmati R Brain white matter damage in SCA1 and SCA2. An in vivo study using voxel-based morphometry, histogram analysis of mean diffusivity and tract-based spatial statistics. Neuroimage (2008) 43:10–9. 10.1016/j.neuroimage.2008.06.036 18672073

[93] Peterburs J Gajda K Koch B Schwarz M Hoffmann KP Daum I Cerebellar lesions alter performance monitoring on the antisaccade task—an event-related potentials study. Neuropsychologia (2012) 50(3):379–89. 10.1016/j.neuropsychologia.2011.12.009 22227094

